# The entanglement between the IUCN Red List and international biodiversity law

**DOI:** 10.1111/cobi.70298

**Published:** 2026-04-22

**Authors:** Rens Claerhoudt

**Affiliations:** ^1^ Tilburg Law School Tilburg University Tilburg The Netherlands

**Keywords:** inequality, international biodiversity law, IUCN Red List, science–law interaction, species bias, *supra‐legality*, derecho internacional para la biodiversidad, desigualdad, interacción ciencia‐legislación, Lista Roja de la UICN, sesgo por especie, supralegalidad

## Abstract

The International Union for Conservation of Nature (IUCN) Red List of Threatened Species (red list) is of fundamental importance for nature conservation and biodiversity protection. I considered the interaction between the red list and international biodiversity law (IBL), the legal regime that aims to protect biodiversity at a global level. The red list is deeply embedded in each of the five global biodiversity treaties. As the most authoritative database on species conservation status, the red list often displays a *supra‐legal* character: its species designations effectively dictate policy makers to act accordingly. In some cases, species designations even create legal facts, a phenomenon I call *auto‐legality*. The entanglement between the red list and IBL makes pertinent the negative critique of the list regarding species bias and interhuman inequalities. The IUCN sets rigorous assessment criteria but refrains from directing who makes the assessments. This results in the red list containing a bias in favor of vertebrate species and a Global North ecovision. Addressing these persistent problems would improve the merit of the close relation between the IUCN Red List and IBL. Species experts must come to terms with the political nature of their work and use it to the benefit of species conservation. Policy makers should invest in a better understanding of the IUCN Red List to optimize its potential for legal species protection.

## Abstract (es)

Entrelazamiento entre la Lista Roja de la UICN y el derecho internacional para la biodiversidad

**Resumen:**

La Lista Roja de Especies Amenazadas de la Unión Internacional para la Conservación de la Naturaleza (UICN) tiene una importancia fundamental para la conservación de la naturaleza y la protección de la biodiversidad. Analicé la interacción entre la Lista Roja y el derecho internacional de la biodiversidad (DIB), el régimen jurídico destinado a proteger la biodiversidad a escala mundial. La lista roja está profundamente arraigada en cada uno de los cinco tratados mundiales sobre biodiversidad. Como base de datos más autorizada sobre el estado de conservación de las especies, la lista roja suele funcionar como un marco supralegal. Cómo designa a las especies obliga con efectividad a los responsables políticos a actuar en consecuencia. En algunos casos, las designaciones de especies llegan incluso a crear hechos jurídicos, un fenómeno que denomino «autolegalidad». El entrelazado entre la lista roja y el DIB hace pertinente la crítica negativa de la lista en lo que respecta al sesgo hacia determinadas especies y a las desigualdades entre los seres humanos. La UICN establece criterios de evaluación rigurosos, pero se abstiene de indicar quién realiza las evaluaciones. Esto da lugar a que la Lista Roja contenga un sesgo a favor de las especies de vertebrados y una visión ecológica del Norte Global. Abordar estos problemas persistentes mejoraría el mérito de la estrecha relación entre la Lista Roja de la UICN y el Derecho Internacional de las Especies. Los expertos en especies deben aceptar la naturaleza política de su trabajo y utilizarla en beneficio de la conservación de las especies. Los responsables políticos deberían invertir en una mejor comprensión de la Lista Roja de la UICN para optimizar su potencial en la protección legal de las especies.

## INTRODUCTION

The devastating rate of anthropogenic biodiversity loss urges fields of science and policymaking to intensify and deepen their collaborations. Particularly interesting is the interaction that takes place between the International Union for Conservation of Nature (IUCN) Red List of Threatened Species (hereafter *red list*) (IUCN, 2025c) and international biodiversity law (IBL) as the international legal regime that addresses the issue of biodiversity loss. The red list is widely considered as the most authoritative and comprehensive data collection on the conservation status of animal, plant, and fungi species (Borgelt et al., 2022; Cowie et al., 2022; Harfoot et al., 2021). Although a red list conservation status determines a species’ extinction risk and not its conservation priority (IUCN, 2012; Miller et al., 2006), the IUCN describes the red list as “a powerful tool to inform and catalyse action for biodiversity conservation and policy change” (IUCN, 2025a).

Despite the transparency of this political ambition and the relevance of the red list in debates on biodiversity conservation—including in recent case law (Court of Justice of the European Union, 2025)—the interaction between the red list and IBL remains a disregarded topic in scholarly literature. I considered the implementation of the red list in the big five of IBL (Trouwborst et al., 2017): the Convention on Biological Diversity (CBD), the Convention on International Trade in Endangered Species of Wild Fauna and Flora (CITES), the Convention on Migratory Species (CMS), the Convention on Wetlands of International Importance especially as Waterfowl Habitat (Ramsar Convention), and the World Heritage Convention (WHC). The recently adopted Agreement under the United Nations Convention on the Law of the Sea on the Conservation and Sustainable Use of Marine Biological Diversity of Areas beyond National Jurisdiction (BBNJ) can be added to this list, but I did not consider it here. Subsequently, I theorize the legal consequences of the entanglement between the red list and IBL, therewith also touching upon scholarly debates on the interplay between science and law at large (Feldman, 2009; Jasanoff, 1997; Sulyok, 2020). This adds to the relevance of scholarly critiques concerning species bias and interhuman inequalities in both the red list and IBL (Armstrong, 2024; Claerhoudt, 2024; Palacio et al., 2023; Tomasini, 2018). With interhuman inequalities, I refer to issues of global (in)justice, such as the disproportional burden that is placed on the Global South by nature conservation efforts (Armstrong, 2024). I argue that addressing these persistent problems improves the merit of the entanglement between the red list and IBL.

## THE BIG FIVE OF IBL AND THE IUCN

### Convention on Biological Diversity

The CBD is the most comprehensive legal instrument of IBL. It covers biodiversity at large, which Article 2 describes as the “diversity within species, between species and of ecosystems.” With nearly universal ratification, the CBD holds a pivotal position in IBL. Although the CBD does not aim to protect specific species, the red list and the IUCN, as such, play important cooperative roles in the CBD. In decisions resulting from the 2022 CBD Conference of Parties (COP), the cooperation between the IUCN and the CBD was mentioned repeatedly, including in the context of knowledge management (CBD COP, 2022c), marine and coastal biodiversity (CBD COP, 2022e), and invasive non‐native species (CBD COP, 2022f). Note, however, that COP decisions are only soft law (i.e., not legally binding).

Succeeding the Aichi Biodiversity Targets, the adoption of the Kunming–Montreal Global Biodiversity Framework (GBF) as “the strategic plan of the Convention and its Protocols” for 2022–2030 has reaffirmed the bond between the CBD and the IUCN (CBD COP, 2022a). Most notably, the IUCN Red List Index functions as headline indicator for the GBF's global goal to halt human induced extinctions as part of its vision for 2050 (CBD COP, 2022b). In addition, the GBF contains 23 targets to be achieved by 2030. Targets 5, 7, and 9 use the index as a component indicator (CBD COP, 2022b). The index also serves as a complementary indicator for six other targets (CBD COP, 2022b). The IUCN is repeatedly mentioned in COP Decision 15/22 (CBD COP, 2022d) as an important party in the Joint Programme of Work on Links between Natural and Cultural Diversity. Similarly, the IUCN calls the CBD “the treaty closest to IUCN's heartland” and describes its role relative to the CBD as “assisting Parties in the revision, update, and development of National Biodiversity Strategies and Action Plans” and “influencing the CBD negotiations to promote conservation” (IUCN, 2025e).

### Convention on International Trade in Endangered Species of Wild Fauna and Flora

Regulating the international import and export of animals and plants, CITES principally functions as a trade agreement. Therefore, the species in its appendices—the lists of species protected under CITES—do not necessarily reflect the conservation status of a species on the red list. The CITES appendices must thus not be expected to closely resemble the red list, although some authors have called for stronger collaboration between the IUCN and CITES (Challender et al., 2023; Gorobets, 2020; Green et al., 2022). A considerable number of CITES Appendix I species, which fall under the strictest trading regime, have not been assessed as threatened by the IUCN (Hutchinson et al., 2021). This does not mean that CITES and the IUCN never cross paths.

Within the CITES framework, the red list can be used to increase the awareness of the dire conservation status of a specific species. Awareness also increases through the cooperation between CITES and the IUCN concerning specific groups of species (e.g., pangolins [CITES COP, 2019]). Furthermore, the presence of 36 representatives of the IUCN during the 2022 CITES COP is indicative of the active participation of the IUCN in CITES developments (CITES, 2022). More generally, the conservation status of a species according to the IUCN must be included in the periodic review of CITES‐listed species (CITES COP, 2022). Red list conservation statuses are sometimes also used to encourage new proposals for CITES appendices. For example, during COP16, the Animal Committee was tasked with studying the red list assessments of Asian snakes (CITES COP, 2013). However, despite their recommendation to consider the conservation statuses of the snakes, especially those that are regularly traded, no new appendix listings of Asian snake species have occurred (CITES Animals Committee, 2014).

### Convention on Migratory Species

The CMS aims to protect migratory species. Importantly, the term *migratory species* must not be understood in a biological but in a jurisdictional sense. Article I(1)(a) of CMS considers species that predictably and cyclically cross nation‐state borders as migratory. The relevance of the red list for CMS is particularly explicit. According to CMS Article III(3)(b), a species may be removed from Appendix I if it is no longer endangered or is not likely to become endangered again due to its removal from Appendix I. The CMS COP (2020) Resolution 13.7 explicitly links the term *endangered* from the convention text to the red list categories of endangered, critically endangered, and extinct in the wild. Hence, as long as a CMS Appendix I species is listed under a red list category, it “should be retained in Appendix I” (CMS COP, 2020). Similarly, these three qualifications make any migratory species “eligible for consideration for listing in Appendix I” (CMS COP, 2020). However, vulnerable or near threatened species are normally not listed in CMS Appendix I (CMS COP, 2020). Appendix II is less strict: species assessed as anything from extinct in the wild to near threatened on the red list are eligible for listing in CMS Appendix II (CMS COP, 2020). Even when a species is assessed as data deficient, a CMS Appendix II listing remains possible, depending on “the merit of any individual Appendix II proposal” (CMS COP, 2020). However, considering such a species for CMS Appendix I “would be exceptional” (CMS COP, 2020).

The latter implies that data‐deficient species are unlikely to be threatened, whereas, in reality, data‐deficient species have a relatively high chance of being threatened (Bland & Böhm, 2016; Borgelt et al., 2022; de Oliveira Caetano et al., 2022; Howard & Bickford, 2014). Next to preventing threatened species from being removed from Appendix I, a conservation status can thus de facto prevent the addition of a species to Appendix I. Paragraph 4g of COP Resolution 13.7 certifies the trust policy makers have in red list assessments by noting that even if no red list assessment is available, “the same principles and percentage changes in populations as the red‐listing process” should be used to provide “an equivalent basis” for the CMS appendix assessment (CMS COP, 2020).

The immediate impact of the red list on CMS does not mean that CMS is simply mimicking the red list. Qualifications serve as bases for the listing of species in appendices, but there remains room for policy makers to influence the decision on the protection regime of each species. In addition, the endangered status of the CMS and the threatened status of the red list do not align. Threatened species on the red list include the categories vulnerable, endangered, and critically endangered. The CMS term *endangered* excludes vulnerable species and includes species that are extinct in the wild (CMS COP, 2020). The latter is remarkable because species that are extinct in the wild may biologically qualify as migrating species, but it is highly unlikely that these species, which live only in captivity, have the option to cyclically and predictably cross jurisdictional borders.

### Convention on Wetlands of International Importance especially as Waterfowl Habitat

The Ramsar Convention aims to protect ecologically significant wetlands through the designation of Ramsar sites. Since the Ramsar Convention drafting process began, the IUCN has been deeply involved in its affairs (Matthews, 1993; Stroud et al., 2022). Not only is the Ramsar Convention Secretariat situated in the headquarters of the IUCN, but the partnership between the organizations is also intensified by a variety of collaborations (IUCN, 2022). These collaborations concern, among other factors, the use of the red list in the activities of the Ramsar Convention. For example, the IUCN's Biodiversity Assessment and Knowledge Team aims to generate data on freshwater species through red list assessments, “particularly as an input into the future expansion of the Ramsar network and for monitoring of biodiversity within existing sites” (IUCN, 2022).

Ramsar COP (2005) Resolution IX.I explicates that red list species assessments that identify threatened species are an important indicator in the designation of Ramsar sites, which contribute to one of the core objectives of the Ramsar Convention, namely, to conserve global wetland biodiversity. Resolution IX.1 thus proposes to directly take into account the red list status of species present at a site when considering its designation as a Ramsar site. This accounting is in line with the criteria for designating sites in the strategic framework and guidelines for the future development of the Ramsar List of Wetlands of International Importance (Ramsar COP, 1999). Criterion 2 holds that “[a] wetland should be considered internationally important if it supports vulnerable, endangered, or critically endangered species or threatened ecological communities” (Ramsar COP, 1999). It adds that the “greatest conservation value will be achieved through the selection of a network of sites providing habitat for rare, vulnerable, endangered, or critically endangered species” (Ramsar COP, 1999). The glossary in Appendix B of Ramsar COP Resolution VII.11 defines the terms *vulnerable*, *endangered*, and *critically endangered* as the IUCN does (Ramsar COP, 1999).

### World Heritage Convention

The WHC encompasses more than 900 cultural heritage sites and over 200 natural heritage sites. Designation as a WHC site often bestows prestige to the relevant state parties (Trouwborst, 2019). The text of the WHC mentions the institutional capacity of the IUCN three times. Article 8(3) says that a representative of the IUCN “may attend the meetings of the World Heritage Committee in an advisory capacity.” Article 13(7) names the IUCN as one of the organizations that the World Heritage Committee can cooperate with for the implementation of its programs. This is reaffirmed in WHC Article 14(2), which prescribes that the director general of the United Nations Educational, Scientific and Cultural Organization (UNESCO), who prepares the committee's documentation and implements the committee's decisions, shall “utiliz[e] to the fullest extent possible the services of (…) the IUCN.”

The *Operational Guidelines for the Implementation of the World Heritage Convention* mentions the IUCN as an advisory body (UNESCO, 2023), in accordance with WHC Article 8(3). This not only ascribes to the IUCN tasks, such as monitoring, reviewing, and providing input, but also asks the IUCN to evaluate sites nominated as potential natural heritage sites (UNESCO, 2023). Although the final decision on the listing of a site remains with the World Heritage Committee, the IUCN carries out the “[e]valuations of natural heritage nomination dossiers” (UNESCO, 2023). Through a strict procedure, a report with recommendations is ultimately submitted to the World Heritage Committee.

The specific designations of sites as World Heritage sites confirm the cooperation between the WHC and the IUCN. Four of the 10 criteria used in assessments of sites of outstanding universal value are applied in the designation of natural World Heritage sites (UNESCO, 2023). For example, the designation of migratory bird sanctuaries along the coast of the Yellow Sea–Bohai Gulf of China is based on criterion (x), which concerns “in‐situ conservation of biological diversity, including those containing threatened species of Outstanding Universal Value from the point of view of science or conservation” (UNESCO, 2023). The relevant decision by the World Heritage Committee explicitly refers to the red list and lists over 20 bird species and their red list status (World Heritage Committee, 2019). Even if not explicitly mentioned, with the IUCN as the main evaluator in “natural heritage nomination dossiers” (UNESCO, 2023), it is unlikely that WHC consideration of endangered and rare species is totally detached from red list data.

## *SUPRA‐LEGALITY* OF THE RED LIST

The above demonstrates that the red list has a direct and broad spillover effect on IBL. Although the IUCN is limited to determining species’ extinction risk (IUCN, 2012; Miller et al., 2006), IBL often connects a species’ red list status to its being a target for conservation. Put differently, the red list imposes standards on policy makers in IBL. To theorize this interaction, I drew on the notion of *supra‐legality* that Johns (2013) developed regarding the relation between international climate change law and the assessment reports of the Intergovernmental Panel on Climate Change (IPCC).

The IPCC assessment reports are conceived as catalysts for legal action, with the idea that “scientists have made decisions about the science or have found things out about the world and international law must act accordingly” (Johns, 2013). In other words, if scientists say X, then policy makers must follow accordingly, often transforming the legally nonbinding into the legally binding. This is typical of what Johns (2013) calls *supra‐legality*: “certain normatively significant practices are so expansive or so endemic as to surpass international law's and lawyers’ grasp or engagement as such” (Johns, 2013). These practices thus occur above or beyond (supra) the law.

Through a culmination of many small interventions and implementations in hard and soft law, the red list has become a recurring factor in the legal instruments of IBL. The formalization of the red list in IBL has only deepened this relationship, ultimately transcending Johns’ (2013) analysis of the *supra‐legality* of the IPCC assessment reports. Policy makers have to translate each separate IPCC assessment report into acceptable legal norms (Johns, 2013). In the case of the red list, the transformation of species assessments into legal norms takes place silently and continuously, changing and affecting the substantive content of various legal instruments. In other words, red list species designations sometimes create legal facts. The designations may not be “a direct consequence of the will of legal persons” (d'Aspremont, 2008), but they undisputably have a legal effect. With the *supra‐legal* IPCC assessment reports, policy makers serve as translators, but in some legal contexts, red list species designations do not even require the approval or translational work of policy makers. Inspired by Johns’ (2013) work, one may grant red list species designations *auto‐legality*, meaning that publication of the scientific results automatically creates legal facts. Consequently, the red list—and the work that species assessors do—has become part of biopolitical decision‐making (Braverman, 2016). Although “[IUCN] listings are meant to inform rather than dictate conservation policy” (Campbell, 2012), in effect, they often achieve both.

## SPECIES BIAS IN THE IUCN RED LIST

A particularly prominent negative criticism on the red list posits that, notwithstanding the rigor of the assessment criteria, the assessment of species is severely imbalanced. For example, of all known fungi and protists, only 0.5% have been assessed (Palacio et al., 2023). Among animals, 90% of the terrestrial vertebrates have been assessed versus only 2% of the known invertebrate species (Palacio et al., 2023). This species bias detracts from the function of the red list as the “barometer of life” (IUCN, 2025b; Stuart et al., 2010) because the extinction risk among various categories may differ.

### Misinterpretation of the data deficient label

Some argue that the unsuitability of the assessment criteria for some categories of species constitutes one explanation for species bias (Bachman et al., 2024; Brummitt et al., 2015; Kwak et al., 2020; Mueller et al., 2022). For example, Cardoso et al. (2011) point out that it is often difficult to gather the required data for invertebrate species for reasons specific to invertebrates. Furthermore, they argue that the design of the assessment criteria makes them poorly applicable to invertebrates.

The data deficient label is commonly misinterpreted. This designation is separate from conservation status categories. It is not a category of threat, but a label that expresses that more data and knowledge are required before an assessment can be undertaken that complies with IUCN standards (IUCN, 2012). Yet the data deficient designation is often misinterpreted as being similar to the least concern category (Parsons, 2016). For policy makers, this means that species do not require extra conservation measures. However, data deficient species appear to be more threatened than other species (Bland & Böhm, 2016; Borgelt et al., 2022; de Oliveira Caetano et al., 2022; Howard & Bickford, 2014). One explanation for this is that there is a causality between the rarity of a species and the deficiency of data; it is much harder to gather sufficient data on a rare species (Roberts et al., 2016). This is further amplified for physiologically small species because they are more difficult to study (Cardoso et al., 2011).

One counterproposal is to automatically qualify data‐deficient species as endangered or critically endangered until sufficient data become available for a species assessment, although, admittedly, this may further invigorate the politicization of species assessments as expressions of conservation priority. With 16,141 out of a total of 94,436 assessed animal species, 6317 of 76,864 assessed plant species, and 290 of 1302 assessed fungi species (IUCN, 2025c), these or similar precautionary measures (e.g., flagging potentially threatened data‐deficient species [Jarić et al., 2016] or providing data‐deficient species precautionary protection measures [Butchart & Bird, 2010; Parsons, 2016]) could have a significant impact, especially because research and conservation programs engaging with data‐deficient species receive substantially less funding (Bland et al., 2015: Jarić et al., 2016). Evidently, these figures represent only a portion of the total known species, which in turn is but a part of the number of total extant species (Larsen et al., 2017).

Although applying the criteria may lead to an underestimation of extinction risks for invertebrates (Cardoso et al., 2011), this does not mean that assessments of species in underrepresented categories cannot be fruitful. In an independent assessment of the host‐specific Manx shearwater flea (*Ceratophyllus [Emmareus] fionnus*), Kwak et al. (2019) found that the assessment of a species based on information about its host, the Manx shearwater (*Puffinus puffinus*), resulted in the species qualifying as vulnerable and thus qualifying as a threatened species based on IUCN criteria. After this study, an IUCN assessment of the Manx shearwater flea was published (not by Kwak et al. [2019]), resulting in its designation as critically endangered (Macadam, 2022). The IUCN assessment showed the potential for assessing invertebrates and particularly parasites, which currently remain severely underrepresented on the red list (Cizauskas et al., 2017; Cowie et al., 2022; Hopkins & Wood, 2026) and in IBL (Claerhoudt, 2024).

### The red list as taxonomy of assessors

The authoritativeness of the red list should not be mistaken for the absolute rationality and objectivity of its publications. The rigorous assessment criteria combined with the fact that “assessments can really be carried out by anyone who has sufficient knowledge of a species” (IUCN, 2025d) makes it probable that only experts on one or several species become assessors. Like the conservationists described by Lorimer (2015), these experts are likely to be “emotional beings,” taking part in “a passionate and embodied practice.” Most of them spend their careers developing expertise on the species they fell in love with through “an epiphany” (Lorimer, 2015). The species that tend to attract prospective students and future experts are usually charismatic species (Lorimer, 2015) or at least invoke a sense of admiration. As a result, species that fall short on these traits are at risk of being neglected by the IUCN and in nature conservation in general (Brambilla et al., 2013; Troudet et al., 2017). This goes for the majority of life‐forms, as particular phenomena, such as plant blindness and disgust toward parasitic animals, indicate (Curtis & de Barra, 2018; Wandersee & Schussler, 1999).

Biologists, ecologists, and taxonomists can perform high‐quality species assessments in line with red list assessments, but their scope of expertise usually does not exceed a handful of species (Rodríguez et al., 2022; Tomasini, 2018). Moreover, none of the red list criteria direct the choice of species for assessment. That choice remains with the individual or collective assessors. Paraphrasing Gaston and May (1992), who described the discipline of taxonomy as a “taxonomy of taxonomists,” red list may therefore be called a taxonomy of assessors. Or, in the words of Tomasini (2018), assessors “select the species to assess and the materials to consult, bring their personal observations and experience, agree on how much is enough data; and define—following IUCN guidelines—which sources of information are deemed reliable.” As I demonstrated above, this has direct consequences for IBL.

The social affect that drives IUCN assessments may result in unequal distribution of assessments between the Global North and the Global South—and therefore in IBL—despite the pluriform contribution of local expertise to species assessments (Tomasini, 2018). Scientists capable of constructing adequate species assessments are predominantly trained in the Global North and work for Global North institutions (Herrera & Maharaj, 2025; Hughes et al., 2023; Miller et al., 2023). Therefore, it is likely that species that attract the interest of experts from the Global North—either through their personal affection or through substantial funding from governments and organizations—are abundant on the red list, giving the red list a Global North‐dominated ecovision (Mabele et al., 2023). The *supra‐legality* of the red list contributes to a legal protection regime of species (including through site protection) that may lead to the neglect of some species and the imposing of an “unfair burden” to states in the Global South (Monsarrat & Svenning, 2022). Similar arguments can be made regarding other inequalities, such as the underrepresentation of women in relevant academic disciplines (Hughes et al., 2023). The red list must thus not only be understood as being the result of human biases but as the merger of various interhuman inequalities. In short, the red list is in fact a list of the species humans—and some humans more than others—care for and whose conservation status and potential extinction affect them for one reason or another.

This view of the red list is in stark contrast with the view of at least some conservationists, who, when interviewed by Braverman (2016), insisted that “the Red List is never political but only scientific.” Such a contention is consistent with the view of science as the creator of absolute, incontestable, objective, and apolitical matters of fact rather than as an inclusive notion of “matters of concern,” as Latour (2004) described over 20 years ago. Remarks by Braverman's interviewees do not hold much truth relative to the red list assessment criteria. Even less convincing is such a belief—for lack of a better word—in the apolitical nature of the red list when considering the decisions that take place outside the application of the assessment criteria. Because anyone can produce a species assessment (IUCN, 2025d)—notwithstanding the obstacles of meeting the rigorous criteria—the decision to assess or not assess a species is always loaded with subjectivity and arbitrariness. The red list is inherently political (Braverman, 2016; Campbell, 2012).

## CHALLENGES AND OPPORTUNITIES

I argue that the *supra‐legality* of the red list offers challenges and opportunities to develop the red list into a more powerful tool in an effort to mitigate biodiversity loss. I considered two actor groups: species experts and international and national policy makers.

Species experts must come to terms with the political nature of their work and use it for the better (Pettorelli et al., 2025). Species assessments matter scientifically and legally. By tying the fate of legal instruments of IBL to the red list, policy makers are less likely to consider unassessed species for legal protection under IBL. Assessed species have a much better chance of being noticed in the realm of the relevant legal instruments, especially when they are threatened. A red list conservation status designation not only elevates certain species to a political or legal status (Braverman, 2016) but also grants them a prioritized status in IBL. In other words, the experts who call for conservation efforts for neglected categories, such as fungi, plants, or parasitic animals (Balding & Williams, 2016; Carlson et al., 2020; Niskanen et al., 2023), may be among the most important addressees of their own messages. The more species assessments published, the more species percolate into the legal protection regimes of IBL. With the development of IUCN Species Survival Commission (SSC) Specialist Groups on underrepresented categories of species, such as parasites, mites, and mollusks, much of the required expertise is already present within the IUCN.

International and national policy makers should invest in a better understanding of the red list. The *supra‐legal* (or even *auto‐legal*) character of the red list enhances the neglect in IBL of a disproportionately large group of species and reinforces interhuman inequalities. Despite the good intentions of building policy on the basis of the best science available, these consequences ultimately weaken one of the core objectives of IBL, namely, to conserve biodiversity (Somsen & Trouwborst, 2021). It is therefore all the more important that policy makers not only learn to understand how the red list functions but also become more aware of its shortcomings, a task that can be supported by experts and collectives situated at the intersection of the IUCN and IBL, such as the Biodiversity Law Specialist Group of the IUCN World Commission on Environmental Law. Simultaneously, policy makers could use the close links of the red list to IBL to improve biodiversity policies, and IBL could incentivize IUCN innovation and knowledge production (Jasanoff, 2004; Verschuuren, 2017). One way to realize this would be to set targets in IBL treaties for state parties to consider the conservation of underrepresented categories of species. This could catalyze an increase in national red list assessments, which may in turn lead to sufficient data and expertise for regional and global assessments of currently understudied species.

## CONCLUSION

My inquiry into the big five of IBL demonstrates that although the red list formally lacks a legally binding character, it is deeply embedded in IBL. Not only is the IUCN heavily involved with the daily operations of the big five, but the IUCN Red List is also often an important tool for policymaking under these treaties. It often serves as a guiding document for policy makers in setting soft law, but sometimes is also used to establish explicit links between legal protection status and red list conservation status. As a result, single‐species assessments may have immediate consequences for the legal status of the assessed species.

These uses justify considering the red list a *supra‐legal* instrument. The list possesses such epistemic and normative authority that it is situated above or beyond the law. In some cases, IBL furthers its relationship with the IUCN by effectively giving red list species assessments an *auto‐legal* character, therewith exceeding and expanding Johns’ (2013) original “vocabulary of non‐legalities.”

With this in mind, acquaintance with negative critiques of the red list is crucial. The assessment criteria have contributed to the construction of an invaluable but biased species list. A side effect of the accessibility of producing species assessments furthers species bias and interhuman inequalities. This combination obstructs the core objectives of both the red list and IBL.

My main argument is not that the status quo must be overthrown but rather that the relevant actors have to adopt a critical stance toward their engagement with the red list and approach the red list for what it inevitably constitutes: a scientific, political, and legal tool that can be used for nature conservation efforts. Against the backdrop of the biodiversity catastrophe, the topic I addressed is important and urgent. Despite the well‐intended objectives of the IUCN and IBL, the close relation between the red list and IBL currently plays a substantial role in leaving the majority of life forms on Earth unnoticed, untouched, and unprotected, for better or for worse.

## References

[cobi70298-bib-0001] Armstrong, C. (2024). Global justice and the biodiversity crisis: Conservation in a world of inequality. Oxford University Press.

[cobi70298-bib-0002] Bachman, S. P. , Brown, M. J. M. , Leão, T. C. C. , Nic Lughadha, E. , & Walker, B. E. (2024). Extinction risk predictions for the world's flowering plants to support their conservation. New Phytologist, 242, 797–808. 38437880 10.1111/nph.19592

[cobi70298-bib-0003] Balding, M. , & Williams, K. J. H. (2016). Plant blindness and the implications for plant conservation. Conservation Biology, 30, 1192–1199. 27109445 10.1111/cobi.12738

[cobi70298-bib-0004] Bland, L. M. , & Böhm, M. (2016). Overcoming data deficiency in reptiles. Biological Conservation, 204, 16–22.

[cobi70298-bib-0005] Bland, L. M. , Collen, B. , Orme, C. D. L. , & Bielby, J. (2015). Predicting the conservation status of data‐deficient species. Conservation Biology, 29, 250–259. 25124400 10.1111/cobi.12372

[cobi70298-bib-0006] Borgelt, J. , Dorber, M. , Høiberg, M. A. , & Verones, F. (2022). More than half of data deficient species predicted to be threatened by extinction. Communications Biology, 5, Article 679. 35927327 10.1038/s42003-022-03638-9 PMC9352662

[cobi70298-bib-0007] Brambilla, M. , Gustin, M. , & Celada, C. (2013). Species appeal predicts conservation status. Biological Conservation, 160, 209–213.

[cobi70298-bib-0008] Braverman, I. (2016). The regulatory life of threatened species lists. In I. Braverman (Ed.), Animals, biopolitics, law: Lively legalities (pp. 19–38). Routledge.

[cobi70298-bib-0009] Brummitt, N. A. , Bachman, S. P. , Griffiths‐Lee, J. , Lutz, M. , Moat, J. F. , Farjon, A. , Donaldson, J. S. , Hilton‐Taylor, C. , Meagher, T. R. , Albuquerque, S. , Aletrari, E. , Andrews, A. K. , Atchison, G. , Baloch, E. , Barlozzini, B. , Brunazzi, A. , Carretero, J. , Celesti, M. , Chadburn, H. , … Nic Lughadha, E. M. (2015). Green plants in the red: A baseline global assessment for the IUCN sampled Red List Index for Plants. PLoS ONE, 10, Article e0135152. 26252495 10.1371/journal.pone.0135152 PMC4529080

[cobi70298-bib-0010] Butchart, S. H. M. , & Bird, J. P. (2010). Data Deficient birds on the IUCN Red List: What don't we know and why does it matter? Biological Conservation, 143, 239–247.

[cobi70298-bib-0011] Caetano, G. H. D. O. , Chapple, D. G. , Grenyer, R. , Raz, T. , Rosenblatt, J. , Tingley, R. , Böhm, M. , Meiri, S. , & Roll, U. (2022). Automated assessment reveals that the extinction risk of reptiles is widely underestimated across space and phylogeny. PLoS Biology, 20, Article e3001544. 35617356 10.1371/journal.pbio.3001544 PMC9135251

[cobi70298-bib-0012] Campbell, L. M. (2012). Seeing red: Inside the science and politics of the IUCN Red List. Conservation and Society, 10, 367–380.

[cobi70298-bib-0013] Cardoso, P. , Borges, P. A. V. , Triantis, K. A. , Ferrández, M. A. , & Martín, J. L. (2011). Adapting the IUCN Red List criteria for invertebrates. Biological Conservation, 144, 2432–2440.

[cobi70298-bib-0014] Carlson, C. J. , Hopkins, S. , Bell, K. C. , Doña, J. , Godfrey, S. S. , Kwak, M. L. , Lafferty, K. D. , Moir, M. L. , Speer, K. A. , Strona, G. , Torchin, M. , & Wood, C. L. (2020). A global parasite conservation plan. Biological Conservation, 250, Article 108596.

[cobi70298-bib-0015] CBD COP . (2022a). Kunming‐Montreal Global Biodiversity Framework (CBD/COP/DEC/15/4). Convention on Biological Diversity.

[cobi70298-bib-0016] CBD COP . (2022b). Monitoring framework for the Kunming‐Montreal Global Biodiversity Framework (CBD/COP/DEC/15/5). Convention on Biological Diversity.

[cobi70298-bib-0017] CBD COP . (2022c). Knowledge management and the clearing‐house mechanism (CBD/COP/DEC/15/16). Convention on Biological Diversity.

[cobi70298-bib-0018] CBD COP . (2022d). Nature and culture (CBD/COP/DEC/15/22). Convention on Biological Diversity.

[cobi70298-bib-0019] CBD COP . (2022e). Conservation and sustainable use of marine and coastal biodiversity (CBD/COP/DEC/15/24). Convention on Biological Diversity.

[cobi70298-bib-0020] CBD COP . (2022f). Invasive alien species (CBD/COP/DEC/15/27). Convention on Biological Diversity.

[cobi70298-bib-0021] Challender, D. W. S. , Cremona, P. J. , Malsch, K. , Robinson, J. E. , Pavitt, A. T. , Scott, J. , Hoffmann, R. , Joolia, A. , Oldfield, T. E. E. , Jenkins, R. K. B. , Conde, D. A. , Hilton‐Taylor, C. , & Hoffmann, M. (2023). Identifying species likely threatened by international trade on the IUCN Red List can inform CITES trade measures. Nature Ecology & Evolution, 7, 1211–1220. 37414949 10.1038/s41559-023-02115-8 PMC10545538

[cobi70298-bib-0022] CITES Animals Committee . (2014). AC27 Doc. 19.3 . Convention on International Trade in Endangered Species.

[cobi70298-bib-0023] CITES COP . (2013). Dec. 16.104 . Convention on International Trade in Endangered Species.

[cobi70298-bib-0024] CITES COP . (2019). Dec. 18.239 . Convention on International Trade in Endangered Species.

[cobi70298-bib-0025] CITES COP . (2022). Res. Conf. 14.8 (Rev. COP19) . Convention on International Trade in Endangered Species.

[cobi70298-bib-0026] Cizauskas, C. A. , Carlson, C. J. , Burgio, K. R. , Clements, C. F. , Dougherty, E. R. , Harris, N. C. , & Phillips, A. J. (2017). Parasite vulnerability to climate change: An evidence‐based functional trait approach. Royal Society Open Science, 4, Article 160535. 28280551 10.1098/rsos.160535 PMC5319317

[cobi70298-bib-0027] Claerhoudt, R. (2024). The legal protection of animal parasites in international biodiversity law. Journal of Environmental Law, 36, 301–322.

[cobi70298-bib-0028] CMS COP . (2020). UNEP/CMS/Resolution 13.7 . Convention on Migratory Species.

[cobi70298-bib-0029] Convention on International Trade in Endangered Species of Wild Fauna and Flora (CITES) . (2022). List of participants of the nineteenth meeting of the Conference of the Parties . https://cites.org/sites/default/files/eng/cop/19/CoP19‐LoP‐final.pdf

[cobi70298-bib-0030] Court of Justice of the European Union . (2025). *MTÜ Eesti Suurkiskjad v Keskkonnaamet* (Case C‐629/23, ECLI:EU:C:2025:429). https://curia.europa.eu/juris/document/document.jsf?text=&docid=301163&pageIndex=0&doclang=en&mode=lst&dir=&occ=first&part=1&cid=9334389

[cobi70298-bib-0031] Cowie, R. H. , Bouchet, P. , & Fontaine, B. (2022). The sixth mass extinction: Fact, fiction or speculation? Biological Reviews, 97, 640–663. 35014169 10.1111/brv.12816 PMC9786292

[cobi70298-bib-0032] Curtis, V. , & de Barra, M. (2018). The structure and function of pathogen disgust. Philosophical Transactions of the Royal Society B: Biological Sciences, 373, Article 20170208. 10.1098/rstb.2017.0208 PMC6000136 29866921

[cobi70298-bib-0033] d'Aspremont, J. (2008). Softness in international law: A self‐serving quest for new legal materials. European Journal of International Law, 19, 1075–1093.

[cobi70298-bib-0034] Feldman, R. (2009). The role of science in law. Oxford University Press.

[cobi70298-bib-0035] Gaston, K. J. , & May, R. M. (1992). Taxonomy of taxonomists. Nature, 356, 281–282.

[cobi70298-bib-0036] Gorobets, A. (2020). Wild fauna conservation: IUCN‐CITES match is required. Ecological Indicators, 112, Article 106091.

[cobi70298-bib-0037] Green, J. , Schmidt‐Burbach, J. , & Elwin, A. (2022). Commercial trade of wild animals: Examining the use of the IUCN Red List and CITES Appendices as the basis for corporate trade policies. Frontiers in Conservation Science, 3, Article 902074.

[cobi70298-bib-0038] Harfoot, M. B. J. , Johnston, A. , Balmford, A. , Burgess, N. D. , Butchart, S. H. M. , Dias, M. P. , Hazin, C. , Hilton‐Taylor, C. , Hoffmann, M. , Isaac, N. J. B. , Iversen, L. L. , Outhwaite, C. L. , Visconti, P. , & Geldmann, J. (2021). Using the IUCN Red List to map threats to terrestrial vertebrates at global scale. Nature Ecology & Evolution, 5, 1510–1519. 34462602 10.1038/s41559-021-01542-9 PMC8560638

[cobi70298-bib-0039] Herrera, M. S. , & Maharaj, G. (2025). Promoting equity between the Global North and Global South in entomological research. Current Opinion in Insect Science, 69, Article 101357. 40044038 10.1016/j.cois.2025.101357

[cobi70298-bib-0040] Howard, S. D. , & Bickford, D. P. (2014). Amphibians over the edge: Silent extinction risk of Data Deficient species. Diversity and Distributions, 20, 837–846.

[cobi70298-bib-0087] Hopkins, S. R. , & Wood, C. L. . (2026). Parasite conservation now: turning knowledge into action. Trends in Parasitology. 10.1016/j.pt.2026.03.009 41935867

[cobi70298-bib-0041] Hughes, A. C. , Than, K. Z. , Tanalgo, K. C. , Agung, A. P. , Alexander, T. , Kane, Y. , Bhadra, S. , Chornelia, A. , Sritongchuay, T. , Simla, P. , Chen, Y. , Chen, X. , Uddin, N. , Khatri, P. , & Karlsson, C. (2023). Who is publishing in ecology and evolution? The underrepresentation of women and the Global South. Frontiers in Environmental Science, 11, Article 1211211.

[cobi70298-bib-0042] Hutchinson, A. , Stephens‐Griffin, N. , & Wyatt, T. (2021). Speciesism and the wildlife trade: Who gets listed, downlisted and uplisted in CITES? International Journal for Crime, Justice and Social Democracy, 11, 191–209.

[cobi70298-bib-0043] International Union for Conservation of Nature (IUCN) . (2012). IUCN Red List Categories and Criteria: Version 3.1 (2nd ed.). Author.

[cobi70298-bib-0044] International Union for Conservation of Nature (IUCN) . (2022). IUCN—Ramsar Collaboration: Supporting the wise use of wetlands. Author.

[cobi70298-bib-0045] International Union for Conservation of Nature (IUCN) . (2025a). Background & history . https://www.iucnredlist.org/about/background‐history

[cobi70298-bib-0046] International Union for Conservation of Nature (IUCN) . (2025b). Barometer of life . https://www.iucnredlist.org/about/barometer‐of‐life 10.1098/rsta.2023.0053 38342209

[cobi70298-bib-0047] International Union for Conservation of Nature (IUCN) . (2025c). IUCN Red List of Threatened Species. Version 2025‐1 . https://www.iucnredlist.org/

[cobi70298-bib-0048] International Union for Conservation of Nature (IUCN) . (2025d). Raw data to red list . https://www.iucnredlist.org/assessment/process 10.1098/rsta.2023.0053 38342209

[cobi70298-bib-0049] International Union for Conservation of Nature (IUCN) . (2025e). UN Convention on Biological Diversity (CBD) . https://www.iucn.org/our‐work/informing‐policy/international‐policy/un‐convention‐biological‐diversity‐cbd

[cobi70298-bib-0050] Jarić, I. , Courchamp, F. , Gessner, J. , & Roberts, D. L. (2016). Potentially threatened: A Data Deficient flag for conservation management. Biodiversity and Conservation, 25, 1995–2000.

[cobi70298-bib-0051] Jasanoff, S. (1997). Science at the bar: Law, science, and technology in America. Harvard University Press.

[cobi70298-bib-0052] Jasanoff, S. (2004). The idiom of co‐production. In S. Jasanoff (Eds.), States of knowledge: The co‐production of science and the social order (pp. 1–12). Taylor and Francis.

[cobi70298-bib-0053] Johns, F. (2013). Non‐legality in international law: Unruly law. Cambridge University Press.

[cobi70298-bib-0054] Kwak, M. L. , Heath, A. C. G. , & Cardoso, P. (2020). Methods for the assessment and conservation of threatened animal parasites. Biological Conservation, 248, Article 108696.

[cobi70298-bib-0055] Kwak, M. L. , Heath, A. C. G. , & Palma, R. L. (2019). Saving the Manx shearwater flea *Ceratophyllus (Emmareus) fionnus* (Insecta: Siphonaptera): The road to developing a recovery plan for a threatened ectoparasite. Acta Parasitologica, 64, 903–910. 31520293 10.2478/s11686-019-00119-8

[cobi70298-bib-0056] Larsen, B. B. , Miller, E. C. , Rhodes, M. K. , & Wiens, J. J. (2017). Inordinate fondness multiplied and redistributed: The number of species on Earth and the new pie of life. Quarterly Review of Biology, 92, 229–265.

[cobi70298-bib-0057] Latour, B. (2004). Why has critique run out of steam? From matters of fact to matters of concern. Critical Inquiry, 30, 225–248.

[cobi70298-bib-0058] Lorimer, J. (2015). Wildlife in the Anthropocene: Conservation after nature. University of Minnesota Press.

[cobi70298-bib-0059] Mabele, M. B. , Kasongi, N. , Nnko, H. , Mwanyoka, I. , Kiwango, W. A. , & Makupa, E. (2023). Inequalities in the production and dissemination of biodiversity conservation knowledge on Tanzania: A 50‐year bibliometric analysis. Biological Conservation, 279, Article 109910.

[cobi70298-bib-0060] Macadam, C. (2022). Ceratophyllus fionnus . The IUCN Red List of Threatened Species 2022: e.T123672975A123674364. International Union for Conservation of Nature.

[cobi70298-bib-0061] Matthews, G. V. T. (1993). The Ramsar Convention on Wetlands: Its history and development. Ramsar Convention Bureau.

[cobi70298-bib-0062] Miller, J. , White, T. B. , & Christie, A. P. (2023). Parachute conservation: Investigating trends in international research. Conservation Letters, 16, Article e12947.

[cobi70298-bib-0063] Miller, R. M. , Rodríguez, J. P. , Aniskowicz‐Fowler, T. , Bambaradeniya, C. , Boles, R. , Eaton, M. A. , Gärdenfors, U. , Keller, V. , Molur, S. , Walker, S. , & Pollock, C. (2006). Extinction risk and conservation priorities. Science, 313, 441–441. 10.1126/science.313.5786.441a 16873627

[cobi70298-bib-0064] Monsarrat, S. , & Svenning, J. (2022). Using recent baselines as benchmarks for megafauna restoration places an unfair burden on the Global South. Ecography, 2022, Article 05795.

[cobi70298-bib-0065] Mueller, G. M. , Cunha, K. M. , May, T. W. , Allen, J. L. , Westrip, J. R. S. , Canteiro, C. , Costa‐Rezende, D. H. , Drechsler‐Santos, E. R. , Vasco‐Palacios, A. M. , Ainsworth, A. M. , Alves‐Silva, G. , Bungartz, F. , Chandler, A. , Gonçalves, S. C. , Krisai‐Greilhuber, I. , Iršėnaitė, R. , Jordal, J. B. , Kosmann, T. , Lendemer, J. , … Dahlberg, A. (2022). What do the first 597 global fungal Red List assessments tell us about the threat status of fungi? Diversity, 14, Article 736.

[cobi70298-bib-0066] Niskanen, T. , Lücking, R. , Dahlberg, A. , Gaya, E. , Suz, L. M. , Mikryukov, V. , Liimatainen, K. , Druzhinina, I. , Westrip, J. R. S. , Mueller, G. M. , Martins‐Cunha, K. , Kirk, P. , Tedersoo, L. , & Antonelli, A. (2023). Pushing the frontiers of biodiversity research: Unveiling the global diversity, distribution, and conservation of fungi. Annual Review of Environment and Resources, 48, 149–176.

[cobi70298-bib-0067] Palacio, R. D. , Abarca, M. , Armenteras, D. , Balza, U. , Dollar, L. J. , Froese, G. Z. L. , Galligan, B. P. , Giordano, A. J. , Gula, J. , Jacobson, A. P. , Jędrzejewski, W. , Khorozyan, I. , Mastretta‐Yanes, A. , Moreno, J. S. , Mudumba, T. , Nana, E. D. , Naveda‐Rodríguez, A. , Negret, P. J. , Crespo, G. O. , … Hughes, A. C. (2023). The global influence of the IUCN Red List can hinder species conservation efforts . Authorea. 10.22541/au.169945445.50394320/v1

[cobi70298-bib-0068] Parsons, E. C. M. (2016). Why IUCN should replace “Data Deficient” conservation status with a precautionary “Assume Threatened” status—A cetacean case study. Frontiers in Marine Science, 3, Article 193.

[cobi70298-bib-0069] Pettorelli, N. , Gaston, K. J. , Barlow, J. , Araújo, M. B. , Bustamante, M. M. D. C. , Chown, S. L. , Diele‐Viegas, L. M. , Laurance, W. F. , Lees, A. C. , Melo, F. P. L. , Milner‐Gulland, E. J. , Pecl, G. , & Sousa‐Pinto, I. (2025). Six actions for ecologists in times of planetary crisis. Nature Ecology & Evolution, 9, 1300–1301. 40481148 10.1038/s41559-025-02759-8

[cobi70298-bib-0070] Ramsar COP . (1999). Resolution VII.11 . Convention on Wetlands of International Importance especially as Waterfowl Habitat.

[cobi70298-bib-0071] Ramsar COP . (2005). Resolution IX.1 . Convention on Wetlands of International Importance especially as Waterfowl Habitat.

[cobi70298-bib-0072] Roberts, D. L. , Taylor, L. , & Joppa, L. N. (2016). Threatened or Data Deficient: Assessing the conservation status of poorly known species. Diversity and Distributions, 22, 558–565.

[cobi70298-bib-0073] Rodríguez, J. P. , Sucre, B. , Mileham, K. , Sánchez‐Mercado, A. , De Andrade, N. , Bezeng, S. B. , Croukamp, C. , Falcato, J. , García‐Borboroglu, P. , González, S. , González‐Ciccia, P. , González‐Maya, J. F. , Kemp, L. , Kusrini, M. D. , Lopez‐Gallego, C. , Luz, S. , Menon, V. , Moehlman, P. D. , Raimondo, D. C. , … Xie, Y. (2022). Addressing the biodiversity paradox: Mismatch between the co‐occurrence of biological diversity and the human, financial and institutional resources to address its decline. Diversity, 14, Article 708.

[cobi70298-bib-0074] Somsen, H. , & Trouwborst, A. (2021). Loss of biosphere integrity (biodiversity loss and extinctions). In D. French & L. J. Kotzé (Eds.), Research handbook on law, governance and planetary boundaries (pp. 222–245). Edward Elgar Publishing.

[cobi70298-bib-0075] Stroud, D. A. , Davidson, N. C. , Finlayson, C. M. , & Gardner, R. C. (2022). Development of the text of the Ramsar Convention: 1965–1971. Marine and Freshwater Research, 73, 1107–1126.

[cobi70298-bib-0076] Stuart, S. N. , Wilson, E. O. , McNeely, J. A. , Mittermeier, R. A. , & Rodríguez, J. P. (2010). The barometer of life. Science, 328, 177–177. 20378803 10.1126/science.1188606

[cobi70298-bib-0077] Sulyok, K. (2020). Science and judicial reasoning: The legitimacy of international environmental adjudication. Cambridge University Press.

[cobi70298-bib-0078] Tomasini, S. (2018). Unpacking the Red List: Use (and misuse?) of expertise, knowledge, and power. Conservation and Society, 16, 505–517.

[cobi70298-bib-0079] Troudet, J. , Grandcolas, P. , Blin, A. , Vignes‐Lebbe, R. , & Legendre, F. (2017). Taxonomic bias in biodiversity data and societal preferences. Scientific Reports, 7, Article 9132. 28831097 10.1038/s41598-017-09084-6 PMC5567328

[cobi70298-bib-0080] Trouwborst, A. (2019). Global large herbivore conservation and international law. Biodiversity and Conservation, 28, 3891–3914.

[cobi70298-bib-0081] Trouwborst, A. , Blackmore, A. , Boitani, L. , Bowman, M. , Caddell, R. , Chapron, G. , Cliquet, A. , Couzens, E. , Epstein, Y. , Fernández‐Galiano, E. , Fleurke, F. M. , Gardner, R. , Hunter, L. , Jacobsen, K. , Krofel, M. , Lewis, M. , López‐Bao, J. V. , Macdonald, D. , Redpath, S. , … Linnell, J. D. C. (2017). International wildlife law: Understanding and enhancing its role in conservation. Bioscience, 67, 784–790. 29599544 10.1093/biosci/bix086 PMC5862276

[cobi70298-bib-0082] UN Educational, Scientific and Cultural Organization (UNESCO) . (2023). *Operational guidelines for the implementation of the World Heritage Convention* (WHC.23/01). Author.

[cobi70298-bib-0083] Verschuuren, J. (2017). Does environmental law encourage or obstruct eco‐innovations? Evidence from case studies in the Netherlands. European Energy and Environmental Law Review, 26, 51–59.

[cobi70298-bib-0084] Wandersee, J. H. , & Schussler, E. E. (1999). Preventing plant blindness. American Biology Teacher, 61, 82–86.

[cobi70298-bib-0085] Whiteman, N. K. , & Parker, P. G. (2005). Using parasites to infer host population history: A new rationale for parasite conservation. Animal Conservation, 8, 175–181.

[cobi70298-bib-0086] World Heritage Committee . (2019). WHC Decision 43 COM 8B.3 . UNESCO.

